# Clinical features and outcomes of focal segmental glomerulosclerosis pathologic variants in Korean adult patients

**DOI:** 10.1186/1471-2369-15-52

**Published:** 2014-03-25

**Authors:** Young Eun Kwon, Seung Hyeok Han, Jeong Hae Kie, Seong Yeong An, Yung Ly Kim, Kyoung Sook Park, Ki Heon Nam, Ah Young Leem, Hyung Jung Oh, Jung Tak Park, Tae Ik Chang, Ea Wha Kang, Shin-Wook Kang, Kyu Hun Choi, Beom Jin Lim, Hyeon Joo Jeong, Tae-Hyun Yoo

**Affiliations:** 1 Department of Internal Medicine, Yonsei University College of Medicine, 50 Yonsei-ro, Seodaemun-Gu, Seoul 120-752, Korea; 2 Department of Pathology, NHIS Medical Center Ilsan Hospital, Goyang-shi, Gyeonggi-do, Korea; 3 Department of Internal Medicine, NHIS Medical Center Ilsan Hospital, Goyang-shi, Gyeonggi-do, Korea; 4 Department of Pathology, Yonsei University College of Medicine, Seoul, Korea

**Keywords:** Focal segmental glomerulosclerosis, Pathology, Outcome

## Abstract

**Background:**

Many studies have shown that clinical characteristics and outcomes differ depending on pathologic variants of focal segmental glomerulosclerosis (FSGS). However, these are not well defined in Asian populations.

**Methods:**

This retrospective study evaluated clinical features and outcomes of pathologic FSGS variants in 111 adult patients between January 2004 and December 2012. Primary outcome was the composite of doubling of baseline serum creatinine concentrations (D-SCr) or onset of end-stage renal disease (ESRD). Secondary outcome included complete (CR) or partial remission (PR).

**Results:**

There were 70 (63.1%), 20 (18.0%), 17 (15.3%), 3 (2.7%), and 1 (0.9%) patients with not-otherwise specified (NOS), tip, perihilar, cellular, and collapsing variants, respectively. At presentation, nephrotic-range proteinuria occurred more commonly in tip lesion than in other variants. The overall 5-year renal survival rate was 76.8%. During a median follow-up of 34.5 months, only 1 (5.0%) patient with a tip lesion reached the composite end point compared to 2 (11.8%) and 12 (17.1%) patients in perihilar and NOS variants, but this difference was not statistically significant in an adjusted Cox model. However, tip lesion was associated with a significantly increased probability of achieving CR (P = 0.044).

**Conclusion:**

Similar to other populations, Korean adult patients with FSGS have distinct clinical features with the exception of a rare frequency of cellular and collapsing variants. Although pathologic variants were not associated with overall outcome, the tip variant exhibited favorable outcome in terms of achieving remission. Further studies are required to delineate long-term outcome and response to treatment of the pathologic variants.

## Background

Focal segmental glomerulosclerosis (FSGS) is one of the most common glomerular diseases leading to end-stage renal disease (ESRD); accounting for 20 to 25% of adult patients undergoing kidney biopsy for evaluation of idiopathic glomerulonephritis (GN) [1]. The estimated incidence of FSGS is 7 per 1 million in the United States [2] and 5.6% of primary glomerular diseases in Korea [3]. A key pathological finding of FSGS on light microscopy is a segmental obliteration involving some portion of glomerular capillaries by the extracellular matrix, but not all glomeruli [4,5]. On electron microscopy, diffuse effacement of foot-process without other abnormalities in the glomerular basement membrane is a prominent finding [5]. It is essentially considered a ‘podocytopathy’, which is associated with various insults directed to podocytes [6]; however, the idiopathic form is most common. The underlying cause of FSGS is uncertain although circulating permeability factors may play a major role in its pathogenesis [7]. The clinical manifestation of FSGS is quite heterogeneous, ranging from asymptomatic presentation with proteinuria and/or microscopic hematuria to heavy proteinuria accompanied by nephrotic syndrome. Of note, FSGS with nephrotic syndrome exhibits a grave prognosis; responsiveness to corticosteroids is not favorable compared to minimal change disease (MCD) and a substantial number of patients with FSGS develop ESRD, especially in African Americans [7].

In 2003, D’Agati, *et al*. proposed 5 histologic variants of FSGS based entirely on light microscopic findings; tip, perihilar, cellular, collapsing, and not-otherwise specified (NOS) [8]. This is known as the Columbia classification, which has gained wide acceptance during the past decade. To date, many investigators have tried to find differences in the clinical characteristics and outcomes of FSGS patients according to these pathologic variants. In general, the collapsing variant has been reported to have worse renal survival rate compared to other variants, while the tip variant shows the best prognosis and high rates of complete remission [9,10]. However, the prevalence of the 5 variants differs depending on race and ethnicity [11]. In fact, collapsing and cellular variants are more prevalent in African Americans than other populations. In contrast, these lesions are relatively uncommon in Indian and Dutch population [11,12]. In addition, there have been few studies to define the clinical characteristics and outcomes according to the Columbia classification involving the East Asian population. Therefore, we conducted a retrospective study to delineate the prevalence of the 5 FSGS variants and their clinical features and outcomes in Korean adult patients with FSGS.

## Methods

### Ethics

This study was approved by the Institutional Review Board (IRB) of Yonsei University Health System Clinical Trial Center (IRB No; 4-2013-0436). This study was a retrospective medical record-based study and the IRB waived the requirement for written consent from the patients.

### Patient selection

**Figure 1:**
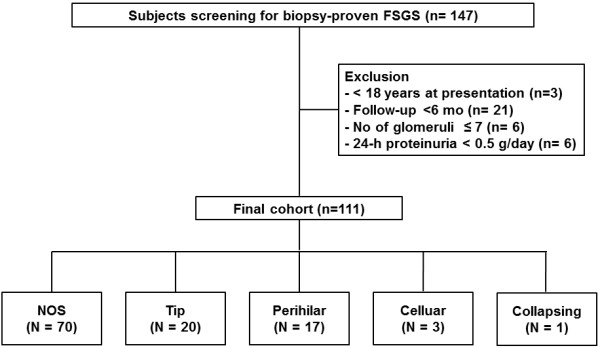
Flow diagram of the study.

A review of the medical records identified 147 patients who were diagnosed with primary FSGS between January 2004 and February 2013 by renal biopsy in Yonsei University Severance hospital and National Health Insurance Service Ilsan hospital in Korea. These patients did not have another glomerular disease or other conditions that were secondarily related to FSGS such as reflux, human immunodeficiency virus (HIV) infection, sickle cell anemia, surgical renal ablation, or solitary kidney. In addition, we confirmed that the patients were not exposed to heroin, lithium, calcineurin-inhibitor, or pamidronate prior to diagnosis. There was no familial FSGS in our medical records. Among these patients, 36 patients were excluded for following reasons; age < 18 years (n = 3), follow-up duration < 6 months (n = 21), inadequate number of glomeruli < 7 (n = 6), and 24-h proteinuria < 0.5 g/day (n = 6). Finally, 111 FSGS patients were included in the analysis (Figure 1).

### Pathologic and clinical data

All biopsied kidney tissues were reviewed by two experienced pathologists, and confirmed as FSGS with segmental scarring of at least one glomerulus and some parts of glomerular tufts according to the definition of FSGS. We classified subtypes of FSGS into tip, perihilar, cellular, collapsing, and not-otherwise specified (NOS) by the Columbia classification [8]. Foot process effacement on electron microscopy (EM) was confirmed by reviewing initial pathologic report.

Demographic and clinical data included age at diagnosis, gender, comorbidities, height, weight, body mass index (BMI), blood pressure, presence of nephrotic syndrome, serum creatinine, estimated glomerular filtration rate (eGFR), serum albumin, total cholesterol, low density lipoprotein (LDL) cholesterol, presence of hematuria, random urine protein-to-creatinine ratio (UPCR), and 24-h urinary protein excretion at the time of biopsy. We calculated eGFR using the CKD-EPI (Chronic Kidney Disease Epidemiology Collaboration) equation [13]. Because 24-h proteinuria was not available at all visits, follow-up data for UPCR were collected. In addition, medications including renin-angiotensin system (RAS) blockers, steroids, and other immunosuppressants were recorded.

We defined hematuria as ≥ 3 red blood cells (RBC) per high power field on urine microscopy. Nephrotic syndrome was defined as 24-h proteinuria > 3.5 g/day, hypoalbuminemia (serum albumin ≤ 3.5 mg/dL), generalized edema and hypercholesterolemia. Nephrotic range proteinuria was defined as 24-h proteinuria > 3.5 g/day or spot urine protein-to-creatinine ratio >3.5 g/g.

### Study endpoints

All patients were followed up until Feb 28, 2013. Primary endpoint was the composite of a doubling of baseline serum creatinine concentration (D-sCr) or onset of ESRD. D-sCr was defined as a sustained, greater than two-fold increase in serum creatinine for at least three consecutive measurements. Point of D-sCr was taken as the first among these measurements. ESRD was defined as initiation of dialysis or receiving transplantation. We also evaluated complete (CR) and partial remission (PR) rates during follow-up. CR was defined as < 0.3 g/g of UPCR and PR was defined as > 50% reduction in proteinuria from baseline amount of proteinuria.

### Statistical analyses

Data analysis was performed using SPSS software for Windows, version 20 (SPSS, Chicago, IL). All variables with normal distribution were presented as mean ± standard deviation. Comparisons were made by Student’s t-tests or one-way ANOVA for continuous variables and by the Chi-square test for categorical variables as appropriate. The Kolmogorov-Smirnov test was used to determine the normality of the distribution of parameters. Data that did not show normal distribution were expressed as median and interquartile range and were compared using the Mann–Whitney test or Kruskal–Wallis test. We estimated the event free renal survival rates of primary end point using the Kaplan-Meier method and compared differences with the log-rank test. Event free survival was calculated from the date of renal biopsy to date of dialysis initiation, kidney transplantation, or last follow-up date. The Cox proportional hazards model was used to identify independent variables for event free survival. The results were expressed in hazard ratios (HR) and 95% confidence intervals (CI). All P-values were two-tailed and values < 0.05 were considered statistically significant.

## Results

### Baseline characteristics

Demographic, clinical and laboratory data by the Columbia classification in 111 patients are shown in Table 1. The frequency of the five variants was 63% (n = 70) in not-otherwise specified (NOS), 18% (n = 20) in tip, 15% (n = 17) in perihilar, 3% (n = 3) in cellular, and 1% (n = 1) in collapsing variants. Patients’ mean age at biopsy was 47.2 years and 39.6% were male. Sixty-three patients (56.8%) had hypertension before FSGS was diagnosed. Mean eGFR was 81.7 mL/min/1.73 m^2^ and median 24-h proteinuria and was 3.02 g/day. Fifty (45.0%) patients developed nephrotic syndrome at presentation (Table 1). When we included the patients with follow-up duration < 6 months (n = 21), and 24-h proteinuria < 0.5 g/day (n = 6), demographic and pathologic characteristics are similar (Additional file 1: Table S1).

**Table 1:** Demogaphic, clinical and laboratory data by FSGS pathologic variants

		**Total**	**NOS**	**Tip**	**Perihilar**	**Cellular**	**Collapsing**	**P†**
N (%)		111	70 (63.1)	20 (18.0)	17 (15.3)	3 (2.7)	1 (0.9)	
Age (years)		47.2 ± 16.2	48.0 ± 15.0	44.8 ± 16.9	45.1 ± 19.0	45.3 ± 18.2	84.0	0.649
Sex	Male (%)	44 (39.6)	29 (41.4)	7 (35.0)	6 (35.3)	2 (66.7)	0 (0.0)	0.818
	Female (%)	67 (60.4)	41 (58.6)	13 (65.0)	11 (64.7)	1 (33.3)	1 (100.0)	
BMI (kg/m^2^)		24.9 ± 3.9	24.8 ± 3.5	25.2 ± 4.5	25.3 ± 4.8	25.7 ± 4.2	23.8	0.837
SBP (mmHg)		133.1 ± 19.5	133.4 ± 19.8	133.8 ± 19.8	130.4 ± 17.3	140.0 ± 34.6	130.0	0.837
DBP (mmHg)		83.2 ± 15.7	83.9 ± 15.8	82.1 ± 11.9	81.4 ± 16.6	90.0 ± 35.3	72.0	0.784
MAP (mmHg)		99.9 ± 16.0	100.4 ± 16.2	99.3 ± 13.5	97.7 ± 15.6	106.8 ± 35.1	91.3	0.813
Hypertension (%)		63 (56.8)	40 (57.1)	9 (45.0)	11 (64.7)	2 (66.7)	1 (100.0)	0.462
Hematuria (%)		43 (38.7)	28 (40.0)	8 (40.0)	6 (35.3)	1 (33.3)	0 (0.0)	0.952
Nephrotic range proteinuria (%)		50 (45.0)	27 (38.6)	14 (70.0)	5 (29.4)	3 (100.0)	1 (100.0)	0.012
Nephrotic syndrome (%)		42 (37.8)	22 (31.4)	12 (60.0)	7 (41.2)	0 (0.0)	1 (100.0)	0.062
Cr (mg/dL)		1.28 ± 1.00	1.26 ± 0.81	0.97 ± 0.48	1.22 ± 0.52	2.11 ± 0.95	1.64	0.277
eGFR (mL/min/1.73 m^2^)		81.7 ± 33.0	81.4 ± 31.7	95.8 ± 34.3	75.8 ± 29.8	46.6 ± 23.4	37.8	0.202
Serum albumin (g/dL)		3.3 ± 1.0	3.4 ± 1.0	2.8 ± 1.0	3.7 ± 0.8	3.3 ± 0.6	1.9	0.018
T- chol (mg/dL)		248.3 ± 104.5	230.0 ± 89.6	340.6 ± 143.7	223.4 ± 52.7	204.0 ± 19.1	241.0	<0.001
LDL-chol (mg/dL)		146.8 ± 80.2	137.5 ± 68.2	215.7 ± 114.1	109.6 ± 39.3	116.5 ± 17.9	162.0	0.002
Proteinuria								
UPCR (g/g)*		3.02 (1.42-5.54)	2.17 (1.33–5.10)	4.67 (2.43-8.26)	1.85 (0.95-4.24)	5.54 (3.94-16.04)	16.67	0.05
24 hr protein (g/day)*		2.90 (1.57-6.32)	2.86 (1.32-5.73)	4.28 (2.07-7.53)	2.32 (0.95-5.20)	4.12 (1.85-12.07)	4.28	0.35
TA-P (g/g)*		1.41 (0.78-3.42)	1.34 (0.73-3.21)	1.67 (0.85-3.46)	1.46 (0.64-4.14)	1.34 (1.11-5.4)	4.85	0.70
Time to renal biopsy (mo)		0 (0 – 2)	0 (0 – 2)	0 (0 – 2)	0 (0 – 2)	0 (0 – 2)	0.0	0.965
Treatment								
RAS blockades (%)		105 (94.6)	66 (94.3)	19 (95.0)	16 (94.1)	3 (100.0)	1 (100.0)	0.991
Steroid (%)		47 (42.3)	23 (32.9)	13 (65.0)	7 (41.2)	3 (100.0)	1 (100.0)	0.035
Responder (n)		25	12	9	2	1	1	
Resistance (n)		22	11	4	5	2	0	
Cyclosporine (%)		25 (22.5)	13 (18.6)	6 (30.0)	4 (23.5)	2 (66.7)	0 (0.0)	0.431
MMF (n)		2	1	0	1	0	0	
Cyclophosphamide (n)		1	1	0	0	0	0	

All data are expressed as mean ± SD or *median and interquartile range.

*Abbreviations:FSGS* focal segmental glomerulosclerosis, *NOS* not otherwise specified, *SBP* systolic blood pressure, *DBP* diastolic blood pressure, *MAP* mean arterial pressure, *S-Cr* serum creatinine, *eGFR* estimated glomerular filtration rate, *T-chol* total cholesterol, *LDL-chol* low-density lipoprotein cholesterol, *UPCR* urine protein-to-creatinine ratio, *TA-P* time-averaged proteinuria, *RAS* renin-angiotensin system, *MMF* mycophenolate mofetil.

†; P-values were obtained from comparisons between NOS, tip, and perihilar variants.

Because there were only three cellular variants and one collapsing variant, we did not include these variants in the statistical analyses. There were no differences in age, sex, BMI, blood pressure, and prior history of hypertension among NOS, tip, and perihilar variants. Nephrotic syndrome occurred more frequently and the amount of proteinuria was higher in tip variant compared to other variants. In addition, total and LDL cholesterol levels were significantly higher and serum albumin levels were significantly lower in patients with tip variant than those with other variants.

### Treatment

Among 50 patients who had nephrotic-range proteinuria over 3.5 g/day at presentation, 47 patients were started on corticosteroid therapy at 1 mg/kg of body weight per day (maximum 80 mg/day). Three patients used cyclosporine as the first line treatment due to osteoporosis. Among those treated with corticosteroids, complete and partial remission were achieved in 17 (36.2%) and 8 (17.0%) patients, respectively. Median duration of corticosteroids treatment was 276 (163–486) days. In the remaining 22 patients who did not respond to corticosteroids, adding cyclosporine resulted in additional CR and PR in one and six patients, respectively.

Most patients (94.6%) were treated with RAS blockers and there was no difference in RAS blockers use among the groups. However, corticosteroids were more commonly prescribed (65.0%) in tip variant compared to other variants (P = 0.035).

### Renal outcomes by the Columbia classification

**Figure 2:**
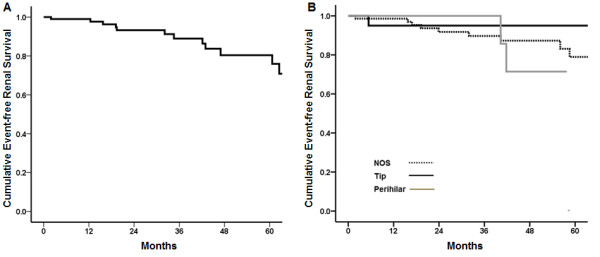
**Kaplan-Meier plots for (A) overall event free renal survival and (B) event free renal survival by FSGS pathologic variants.** Overall 5-renal survival rate was 76.8%. There were no significant difference in renal survival rates between patients with NOS, perihilar, and tip variants.

Median follow up duration was 34.5 months and twenty-seven patients were followed up more than 5 years. Sixteen (14.4%) patients reached the composite of D-sCr or ESRD and the overall 5-year event-free renal survival rate was 76.8% (Figure 2A). Twelve (17.1%) patients with NOS variant reached the composite primary outcome compared to 2 (11.8%) with perihilar variant and 1 (5.0%) with tip variant (Table 2, P = 0.370). A Kaplan-Meier curve also showed that overall renal survival rates were not statistically significant between patients with NOS, tip, and perihilar variants (Figure 2B, P = 0.616). Multivariable analysis after adjustment of age, sex, hypertension, eGFR, proteinuria, and immunosuppression showed no difference in the development of primary outcome among the three groups (Table 3). Among three patients with cellular variant, two patients showed kidney impairment at presentation (eGFR of 24.6 and 36.2 mL/min/1.73 m^2^, respectively) and one reached the composite outcome. At two patients with cellular variant showed UPCR 16.04 and 3.94 g/g at the time of biopsy, UPCR were reduced to 2.84 and 1.93 g/g after treatment of corticosteroid or cyclosporine, respectively. One patient with collapsing variant presented nephrotic syndrome and exhibited an eGFR of 38 mL/min/1.73 m^2^ and UPCR of 16.7 g/g at the time of biopsy. Twelve-month corticosteroid treatment resulted in reduction of UPCR to 1.43 g/g and improvement of eGFR to 38 mL/min/1.73 m^2^ in this patient.

**Table 2:** Renal outcomes by FSGS pathologic variants

	**NOS**		**Tip**		**Perihilar**	
	**N (%)**	**/1000 pys**	**N (%)**	**/1000 pys**	**N (%)**	**/1000 pys**
Composite*	12 (17.1%)	47.6	1 (5.0%)	17.8	2 (11.8%)	45.2
D-SCr	12 (17.1%)	47.6	1 (5.0%)	17.8	2 (11.8%)	45.2
ESRD	8 (11.4%)	31.3	1 (5.0%)	17.6	0 (0.0%)	0.0
CR or PR	38 (54.3%)	337.5	16 (80.0%)^†, ‡^	678.0	7 (41.2%)	220.8
CR	16 (22.9%)	84.7	10 (50.0%)^§, #^	305.8	3 (17.6%)	74.1
PR	22 (31.4%)	153.8	6 (30.0%)	155.8	4 (23.5%)	112.4
Relapse	8 (11.4%)		3 (15.0%)		1 (5.9%)	

*Composite outcome was a doubling of the baseline serum creatinine concentration or ESRD.

†, P = 0.038 vs. NOS; ^‡^, P = 0.015 vs. perihilar; ^§^, P = 0.018 vs. NOS; ^#^, P = 0.040 vs. perihilar.

*Abbreviations:NOS* not-otherwise specified, *D-SCr* doubling of the baseline creatinine levels, *ESRD* end-stage renal disease, *CR* complete remission, *PR* partial remission, pys patient-years.

Footnote; Thirty-five patients achieved partial remission. Thirty-two cases are included in Table 2 and the remaining 3 cases are two patients with cellular variant and a patient with collapsing variant.

**Table 3:** Multivariable Cox regression analyses for the composite outcome

	**Model 1**		**Model 2**		**Model 3**	
	**HR (95% CI)**	**P-value**	**HR (95% CI)**	**P-value**	**HR (95% CI)**	**P-value**
Sex (vs. female)	0.658 (0.189 – 2.292)	0.511	0.821 (0.215 – 3.132)	0.773	0.741 (0.175 – 3.149)	0.685
Age (per 1 year increase)	0.952 (0.914 – 0.992)	0.020	0.950 (0.911 – 0.991)	0.017	0.945 (0.902 – 0.991)	0.019
HTN (vs. no)	0.675 (0.184 – 2.476)	0.553	0.793 (0.194 – 3.242)	0.746	0.663 (0.138 – 3.198)	0.609
TAP (per 1 g/g increase)	1.887 (1.399 – 2.546)	<0.001	1.881 (1.392 – 2.543)	<0.001	1.979 (1.382 – 2.832)	<0.001
eGFR (per 1 ml/min/1.73 m^2^ increase)	0.955 (0.932 – 0.979)	<0.001	0.951 (0.923 – 0.978)	0.001	0.952 (0.926 – 0.978)	<0.001
Pathologic variants						
NOS	–	–	Reference		Reference	
Perihilar	–	–	1.054 (0.189 – 5.862)	0.952	1.019 (0.183 – 5.664)	0.983
Tip	–	–	1.699 (0.160 – 18.052)	0.660	1.657 (0.162 – 16.949)	0.670
Immunosuppression (vs. no)			–	–	0.622 (0.120 – 3.225)	0.572

*Abbreviations:HTN* hypertension, *eGFR* estimated glomerular filtration rate, *TAP* time-averaged proteinuria.

Model 1: sex, age, hypertension, TAP, and eGFR.

Model 2: Model 1 + pathologic variants.

Model 3: Model 2 + immunosuppression.

### CR or PR by the Columbia classification

**Figure 3:**
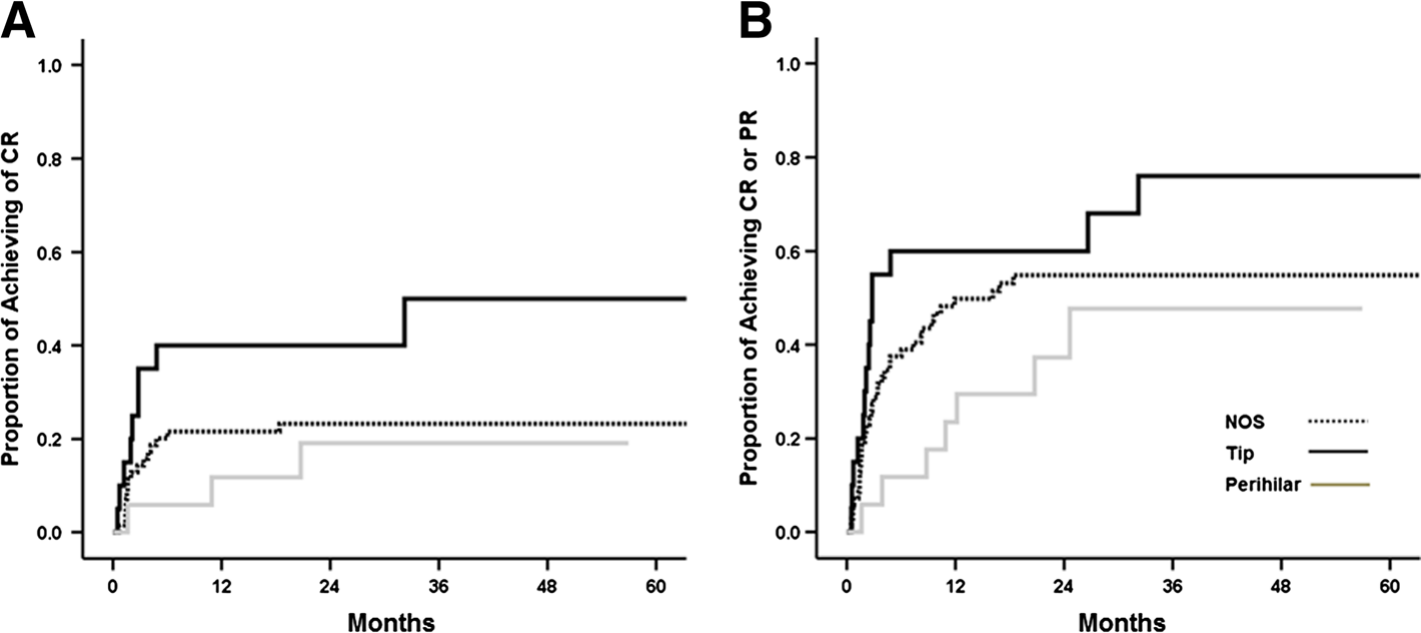
**Kaplan-Meier plots for (A) complete remission (CR) and (B) complete or partial remission (PR).** Patients with tip lesion had higher probability of achieving CR and CR or PR.

In terms of remission rates, CR and PR were achieved in 29 (26.1%) and 35 (31.5%) patients, respectively. CR or PR was attained in 16 (80.0%) patients with tip variant compared to 38 (54.3%) with NOS variant (P = 0.038) and 7 (41.2%) with perihilar variant (P = 0.015) (Table 2). In addition, CR was more frequently achieved in patients with tip variant than those with NOS (P = 0.018) or perihilar (P = 0.040) variants. A higher rate of PR was also observed in patients with tip variant compared to those with other variants, but did not reach statistical significance. Kaplan-Meier plots also produced the same results (Figure 3A and B). In unadjusted Cox models, tip variant was associated with a significantly increased probability of achieving CR (HR, 2.616; 95% CI, 1.184-5.778; P = 0.017) and CR or PR (HR, 1.809; 95% CI, 1.007-3.250; P = 0.047). Multivariable analysis adjusted for age, sex, eGFR, proteinuria, and immunosuppression showed that tip variant conferred a 2.4-fold higher probability of achieving CR (P = 0.044, Table 4). A similar association was also observed when CR or PR was entered together as a dependent outcome variable, but the result was not statistically significant.

**Table 4:** Unadjusted and adjusted Cox models for remission

	**Unadjusted**		Adjusted*	
	**HR (95% CI)**	**P-value**	**HR (95% CI)**	**P-value**
CR				
Pathologic variants				
NOS	Reference		Reference	
Perihilar	0.736 (0.214 – 2.534)	0.627	0.963 (0.262 – 3.535)	0.954
Tip	2.616 (1.184 – 5.778)	0.017	2.417 (1.025 – 5.703)	0.044
CR or PR				
Pathologic variants				
NOS	Reference		Reference	
Perihilar	0.616 (0.274 – 1.384)	0.241	0.691 (0.303 – 1.580)	0.381
Tip	1.809 (1.007 – 3.250)	0.047	1.632 (0.870 – 3.061)	0.127

*Adjusted for age, sex, eGFR, proteinuria, and immunosuppression.

*Abbreviations:CR* complete remission, *PR* partial remission, *NOS* not otherwise specified.

## Discussion

In this study, we sought to delineate clinical features and outcomes according to the Columbia classification in 111 Korean adult patients with primary FSGS. We showed that cellular and collapsing variants were uncommon in our cohort and overall outcome was not affected by pathologic variants. However, nephrotic syndrome was the most common in tip variant, which exhibited favorable outcome in terms of achieving remission. The present study provided distinct clinical features of FSGS in the Korean population for the first time and reinforced the findings of previous studies indicating that pathologic variants of FSGS may have different prognostic implications.

As aforementioned, the Columbia classification has been used worldwide in patients with FSGS. However, many studies have clearly shown that the relative frequency of the five variants varies depending on race and ethnicity [4,9,11,12,14-17]. It is well known that collapsing and cellular variants are more common in African Americans than other populations, while whites are more likely to have tip variant [9,14]. However, collapsing and cellular variants were not common in the present study. This finding is corroborated by other previous studies showing a low frequency of collapsing and cellular variants in non-African American populations [11,12]. To date, there have been few studies to evaluate the prevalence of the five pathologic variants by the Columbia classification in Asian populations. In keeping with our findings, NOS variant was the most common and collapsing variant was uncommon in an Indian study and two Chinese studies [11,18,19]. However, four Asian studies were not in accordance with the frequency of cellular variant. Our cohort showed the lowest (2.7%) frequency, while it was the highest for the Chinese studies (14.4% and 25.5%) and intermediate between the two populations for the Indian study (8.0%). Interestingly, there was a substantial discrepancy in the frequency of cellular variant between Korean and Chinese populations. Moreover, the two Chinese studies reported a 10% difference in the frequency although they were the same population. Of note, there may be misclassification of cellular variant as tip variant because cellular lesions can exist within the tuft in tip variants and intracapillary expansile foam cells can be observed in both variants depending on location; either at the polar domain in tip variants or at any other location in the cellular variants. In fact, Stokes *et al.*[17] emphasized the importance of adequate sampling and sectioning because about 30% of cellular variants were reclassified as tip variants by deeper sectioning of the biopsy. On the other hand, there has been controversy about whether tip and cellular variants may be two stages of a single entity identified at different points of time [11,17]. Due to the retrospective nature of the study, it is unknown whether additional deeper tissue sectioning might result in more identification of tip variants in the Chinese studies. Nevertheless, our findings were consistent with most previous studies showing that cellular variant is uncommon. Further studies involving other Asian populations are warranted to elucidate whether cellular variant is as common in Asian populations as in African Americans.

In general, collapsing variant has the worst prognosis among the five variants, whereas tip variant has the best prognosis. Cellular variant shows an intermediate prognosis between the two variants. Unfortunately, the present study did not confirm these observations because of the limited number of collapsing and cellular variants. Two patients with cellular variant and one patient with collapsing variant had substantial amounts of proteinuria and kidney impairment at presentation. This finding suggests that clinical outcomes of these two variants may not be favorable, and rare frequency of these two variants may draw a favorable prognosis. However, long-term follow-up studies with a larger sample size are required to determine the prognosis.

A number of studies have reported that tip variant has clinical features similar to that of minimal change disease and its responsiveness to corticosteroids is favorable; thus, tip variant is considered to have good prognosis [10,20]. In line with this, in the present study, tip variant was significantly associated with achieving CR or PR. In addition, only 1 (5.0%) patient with tip variant reached the composite outcome compared to 12 (17.1%) patients with NOS variant and 2 (11.8%) patients with perihilar variant. However, this difference was not statistically significant possibly because of the small number of patients. Of note, previous studies compared tip variant with collapsing or cellular variants [10,14], which are known to be associated with poor outcome. As aforementioned, in our cohort, these two variants were very rare; thus, detailed analysis to prove prognostic superiority of tip variant to other variants was not feasible. Because NOS and perihilar variants generally present subnephrotic range proteinuria and less severe clinical features compared to collapsing variant, it is possible that tip variant may not confer an advantage in renal outcome over NOS or perihilar variants depending on the characteristics of the study subjects. In fact, two previous studies did not demonstrate that renal outcome of tip variant is better than that of NOS variant [10,14]. Nevertheless, given the significant correlation between remission rate and renal survival, it can be expected that tip variant may have favorable long-term outcome.

Perihilar variant is generally considered secondary FSGS. In the present study, patients with this variant comprised 15.3% of all patients and had no evidence of reflux nephropathy, sickle cell anemia, surgical ablation, or renal agenesis. Eleven (64.7%) patients were diagnosed with hypertension before renal biopsy, and some of them showed histologic features of hypertensive nephrosclerosis, which might contribute to adaptive change in glomerular capillaries. In our cohort, obesity was unlikely to be associated with perihilar variant because mean BMI was 25.3 kg/m^2^, which was not different from patients with other variants. Nephrotic syndrome developed in 41.2% of patients with perihilar variant. In addition, the clinical outcome of these patients was favorable because 5-year renal survival rate was 66.7% (Figure 2B) and only two (11.8%) patients reached the composite outcome during follow-up. Our findings were consistent with the results of previous studies showing that the frequency of nephrotic syndrome in perihilar variant varies from 25% to 55% [9,12] and 5-year renal survival rate was reported up to 55% [12].

Our study has some limitations. First, this is a retrospective study with a small sample size. In particular, a small number of patients with cellular and collapsing variants rendered statistical analysis unavailable. Thus, we could not analyze these patients to prove that collapsing or cellular variant exhibit the worst prognosis as previously reported. For the same reason, tip variant was not associated with a significantly decreased risk of reaching the composite outcome. Second, we confirmed foot process effacement on EM reviewed initial pathologic reports due to lack of available EM photography in some patients. Third, the retrospective nature of the study did not clearly suggest a therapeutic approach by histologic variants. In fact, in many cases, physicians usually decide whether or not to treat with immunosuppression depending on clinical indicators such as heavy proteinuria or rapid deterioration of kidney function. In addition, there has been a concern about the usefulness of pathologic findings in predicting future outcome. Several studies showed that response rate to treatment in collapsing FSGS was not as poor as expected, ranging as high as 40 to 64% [1,12]. In addition, Chun *et al*., reported a 92% remission rate in patients with celluar lesions involving < 20% of glomeruli, compared to only 33% in patients with cellular lesions in ≥ 20% of glomeruli [14], suggesting that cellular lesions per se do not universally portend a bad prognosis. In line with this notion, in the present study, entering CR or PR was significantly associated with a decreased risk of reaching the composite outcome (HR, 0.03; 95% CI, 0.002-0.594; P = 0.021, data not shown), while pathologic variants were not. Finally, there was relatively small number of patients presenting with nephrotic syndrome in this study. This is partly attributed to a lack of patients with collapsing and cellular variants in our study. In general, these two variants show nephrotic syndrome. In contrast, subnephrotic proteinuria is more common in patients with NOS and perihilar variants, which comprised 78.4% of our cohort. Such characteristics of our cohort may explain the low prevalence of nephrotic syndrome in this study.

## Conclusion

In conclusion, the present study might help illuminate common clinical features of FSGS with a rare frequency of cellular and collapsing variants in the Korean population. Although pathologic variants were not associated with overall outcome, tip variant showed favorable outcome in terms of achieving remission. However, since present study includes very small number of patients, further studies with a larger sample size are required to delineate long-term outcome and response to treatment of the pathologic variants.

## Competing interest

The authors declare that they have no competing interests.

## Authors’ contributions

YEK, SHH and THY conceived and designed the experiments. JHK, SYA, YLK, KSP, KHN and AYL analyzed the data. YEK, SHH and THY wrote the paper. HJO, JTP, TIC and EWK carried out data collection. SWK, KHC, BJL and HJJ participated in the interpretation of data. All authors read and approved the final manuscript.

## Pre-publication history

The pre-publication history for this paper can be accessed here:

http://www.biomedcentral.com/1471-2369/15/52/prepub

## Supplementary Material

Additional file 1: Table S1 Demogaphic, clinical and laboratory data by FSGS pathologic variants in patients including less proteinuria and shorter follow up duration. Click here for file
