# Supplementary material for: Clinical features and outcomes of focal segmental glomerulosclerosis pathologic variants in Korean adult patients
Source: BMC Nephrol. 2014 Mar 25;15:52. doi: 10.1186/1471-2369-15-52 (PMC4230406; doi:10.1186/1471-2369-15-52)
Supplement: Additional file 1: Table S1 — Demogaphic, clinical and laboratory data by FSGS pathologic variants in patients including less proteinuria and shorter follow up duration. [file 1471-2369-15-52-S1.docx]

**Additional file 1: Table S1. Demogaphic, clinical and laboratory data by FSGS pathologic variants in patients including less proteinuria and shorter follow up duration**

|  | | **Total** | **NOS** | **Tip** | **Perihilar** | **Cellular** | **Collapsing** | **P†** |
| --- | --- | --- | --- | --- | --- | --- | --- | --- |
| N (%) | | 138 | 84 (60.9) | 26 (18.8) | 20 (14.5) | 5 (3.6) | 3 (2.2) |  |
| Age (years) | | 47.1 ± 17.3 | 48.8 ± 15.8 | 44.2 ± 18.9 | 43.9 ± 19.4 | 47.0 ± 18.9 | 46.7 ± 32.6 | 0.694 |
| Sex | Male (%) | 62 (44.9) | 37 (44.0) | 13 (50.0) | 7 (35.0) | 4 (80.0) | 1 (33.3) | 0.442 |
|  | Female (%) | 76 (55.1) | 47 (56.0) | 13 (50.0) | 13 (65.0) | 1 (20.0) | 2 (66.7) |  |
| BMI (kg/m^2^) | | 24.7 ± 3.8 | 24.5 ± 3.3 | 24.6 ± 4.5 | 25.0 ± 4.7 | 25.0 ± 3.1 | 25.0 ± 4.9 | 0.275 |
| SBP (mmHg) | | 134.6 ± 22.4 | 133.8 ± 19.1 | 134.6 ± 18.4 | 128.5 ± 17.4 | 168.6 ± 65.7 | 140.0 ± 10.0 | 0.122 |
| DBP (mmHg) | | 83.5 ± 14.7 | 83.7 ± 15.0 | 83.3 ± 11.5 | 81.2 ± 15.2 | 87.8 ± 25.5 | 89.0 ± 14.9 | 0.857 |
| MAP (mmHg) | | 100.4 ± 16.1 | 100.4 ± 15.5 | 100.4 ± 12.6 | 97.0 ± 14.6 | 114.7 ± 38.6 | 106.0 ± 13.1 | 0.267 |
| Hypertension (%) | | 74 (53.6) | 49 (58.3) | 9 (34.6) | 12 (60.0) | 3 (60.0) | 1 (33.3) | 0.246 |
| Hematuria (%) | | 61 (44.2) | 39 (46.4) | 12 (46.2) | 7 (35.0) | 2 (40.0) | 1 (33.3) | 0.986 |
| Nephrotic range proteinuria (%) | | 63 (45.7) | 33 (39.3) | 15 (57.7) | 7 (35.0) | 5 (100.0) | 3 (100.0) | 0.010 |
| Cr (mg/dL) | | 1.32 ± 1.11 | 1.28 ± 1.00 | 0.98 ± 0.42 | 1.18 ± 0.52 | 3.72 ± 3.01 | 1.94 ± 1.51 | 0.001 |
| eGFR (mL/min/1.73m^2^) | | 77.6 ± 27.2 | 80.4 ± 28.6 | 90.6 ± 30.7 | 82.9 ± 26.5 | 43.2 ± 30.1 | 59.1 ± 55.8 | 0.021 |
| Serum albumin (g/dL) | | 3.3 ± 1.1 | 3.4 ± 1.0 | 3.1 ± 1.2 | 3.5 ± 0.9 | 2.9 ± 0.8 | 2.3 ± 1.4 | 0.272 |
| T- chol (mg/dL) | | 246.9 ± 104.4 | 225.8 ± 88.6 | 301.9 ± 144.9 | 248.3 ± 83.2 | 266.6 ± 87.9 | 324.0 ± 97.2 | 0.012 |
| LDL-chol (mg/dL) | | 151.2 ± 88.4 | 130.7 ± 68.1 | 206.5 ± 114.9 | 145.8 ± 92.2 | 176.9 ± 108.8 | 246.6 ± 119.6 | 0.023 |
| Proteinuria | |  |  |  |  |  |  |  |
| UPCR (g/g) * | | 3.47(1.30-7.19) | 2.41(1.23–6.72) | 4.77(2.03-9.01) | 1.68(1.05-4.50) | 6.53(4.74-11.64) | 14.65(12.62-16.67) | 0.035 |
| 24hr protein (g/day) * | | 3.13(1.57-7.13) | 2.88(1.31-6.70) | 4.09(1.86-7.47) | 2.33(1.60-6.04) | 3.50(3.20-4.12) | 11.84(4.28-19.39) | 0.607 |

All data are expressed as mean ± SD or *median and interquartile range.

Abbreviations: FSGS, focal segmental glomerulosclerosis; NOS, not otherwise specified; SBP, systolic blood pressure; DBP, diastolic blood pressure; MAP, mean arterial pressure; S-Cr, serum creatinine; eGFR, estimated glomerular filtration rate; T-chol, total cholesterol; LDL-chol, low-density lipoprotein cholesterol; UPCR, urine protein-to-creatinine ratio.

†; P-values were obtained from comparisons among pathologic variants.
